# Primordial Capsid and Spooled ssDNA Genome Structures Unravel Ancestral Events of Eukaryotic Viruses

**DOI:** 10.1128/mbio.00156-22

**Published:** 2022-07-20

**Authors:** Anna Munke, Kei Kimura, Yuji Tomaru, Han Wang, Kazuhiro Yoshida, Seiya Mito, Yuki Hongo, Kenta Okamoto

**Affiliations:** a The Laboratory of Molecular Biophysics, Department of Cell and Molecular Biology, Uppsala University, Uppsala, Sweden; b Department of Biological Resource Science, Faculty of Agriculture, Saga University, Saga, Japan; c Fisheries Technology Institute, Japan Fisheries Research and Education Agency, Hatsukaichi, Hiroshima, Japan; d Graduate School of Agriculture, Saga University, Saga, Japan; e Bioinformatics and Biosciences Division, Fisheries Resources Institute, Japan Fisheries Research and Education Agency, Fukuura, Kanazawa, Yokohama, Kanagawa, Japan; Boston University School of Medicine

**Keywords:** algae virus, bacilladnavirus, capsid protein evolution, diatom virus, genome structure, horizontal gene transfer, nodavirus, ssDNA virus

## Abstract

Marine algae viruses are important for controlling microorganism communities in the marine ecosystem and played fundamental roles during the early events of viral evolution. Here, we have focused on one major group of marine algae viruses, the single-stranded DNA (ssDNA) viruses from the *Bacilladnaviridae* family. We present the capsid structure of the bacilladnavirus *Chaetoceros tenuissimus* DNA virus type II (CtenDNAV-II), determined at 2.4-Å resolution. A structure-based phylogenetic analysis supported the previous theory that bacilladnaviruses have acquired their capsid protein via horizontal gene transfer from a ssRNA virus. The capsid protein contains the widespread virus jelly-roll fold but has additional unique features; a third β-sheet and a long C-terminal tail. Furthermore, a low-resolution reconstruction of the CtenDNAV-II genome revealed a partially spooled structure, an arrangement previously only described for dsRNA and dsDNA viruses. Together, these results exemplify the importance of genetic recombination for the emergence and evolution of ssDNA viruses and provide important insights into the underlying mechanisms that dictate genome organization.

## INTRODUCTION

Marine algae viruses prevail massively in the oceans and greatly affect the global ecosystem by causing mortality and lysis of microbial communities, releasing organic carbon and other nutrients back into the environment (the “viral shunt”), thereby affecting the dynamics of algal blooms, the global oxygen level, and the marine nutrient and energy cycling (1–3). The viruses typically have a very narrow host range, thus causing host-specific mortality and control of algae host populations (4).

Virus capsid evolution is an increasingly growing research field that relies both on sequence and structural analysis, as well as on computational approaches (5–12). These studies range from narrow comparisons between specific virus groups (6, 8, 10, 11) to large-scale examinations across the entire virosphere (7) and have contributed to the identification of unique structural traits among certain viruses (8, 10–12) and the revelation of evolutionary relationships between seemingly unrelated viruses (5, 6, 9, 13). Moreover, a defined number of viral lineages have been identified based on capsid protein folds (5, 9), which were originally acquired from cells on multiple independent occasions (7). Since unicellular marine organisms were the earliest eukaryotes on earth, they were presumably host of the most ancient viruses (14, 15). Present-day viruses infecting unicellular organisms, such as unicellular algae, therefore likely retain genetic and structural characteristics from their ascendants (8, 16) and are consequently an essential group of viruses for interrogating viral capsid evolution.

Bacilladnaviruses is one of the major group of viruses infecting eukaryotic algae. They carry a circular ssDNA genome of ~6 kb, which is partially double stranded (~700 to 800 bp) and encodes three proteins; one coat protein, one replication-associated protein (Rep), and a third protein with unknown function (17). Until recently, bacilladnaviruses have been included in the informal group CRESS DNA viruses (for circular Rep-encoding ssDNA viruses), but this group has now formed the phylum *Cressdnaviricota* based on their relatively conserved Rep protein (18). The Rep protein, suggested to be evolved from bacterial plasmids (19), has two functional domains, a His-hydrophobic-His endonuclease and a superfamily 3 helicase, and is involved in the virus genome replication, which is carried out by a rolling-circle mechanism (20). Contrary, the capsid proteins of CRESS DNA viruses are very diverse and have presumably been acquired from RNA viruses on multiple independent occasions (6, 13, 21–23). Specifically, bacilladnaviruses were suggested to have acquired their capsid proteins through horizontal gene transfer (HGT) from an ancestral noda-like virus (6). This is not an unreasonable scenario considering the prevalence of noda-like viruses in the aquatic environment (24). However, structural evidence has until now been pending.

Here, we present 3D reconstructions that reveal both the capsid and genome organizations of the bacilladnavirus *Chaetoceros tenuissimus* DNA virus type II (CtenDNAV-II) (17). An atomic model of the capsid protein could be constructed from the 2.4-Å resolution capsid structure. Structure-based phylogeny was used to demonstrate that the capsid of bacilladnaviruses indeed is structurally more similar to capsids of RNA viruses than to those of other ssDNA viruses, corroborating the HGT theory in early virus evolution. In addition, a low-resolution density map of the ssDNA genome suggests a partially spooled genome packaging mechanism, which has previously only been described for dsRNA and dsDNA viruses.

## RESULTS AND DISCUSSION

### Summary of structure determination.

The structure of the CtenDNAV-II viron was determined using cryo-electron microscopy (cryo-EM). The capsid was reconstructed by imposing icosahedral symmetry (I4) and using 33,507 particles to an overall resolution of 2.4 Å using the “gold standard” Fourier shell correlation (FSC) 0.143 criterion (25, 26) (Fig. S1C). The local resolution of the capsid reconstruction was distributed between 2.3 and 9.1 Å and estimated using ResMap (27) (Fig. S1D). An atomic model of the capsid was built, refined, and validated according to the cryo-EM map. During 3D classification, it became apparent that possibly also the capsid interior possessed higher order structure (Fig. S2A), yet no density was observed in the final icosahedrally averaged high-resolution reconstruction. However, when employing a previously described method of subtracting the contribution of the capsid (28), followed by a number of 3D classification steps, it became apparent that parts of the genome formed an outer layer. Using a subset of 21,559 particles and without imposing any symmetry (C1), the outer genome layer could be reconstructed to 13 Å (FSC = 0.143 criterion). Figure S2B shows the FSC curves of the C1 reconstruction, where also the resolution criterion FSC = 0.5 (20 Å) is indicated as a comparison. An additional subtraction of the outer genome layer was attempted to reconstruct the core; however, the even lower resolution of the resulting reconstruction made it uninterpretable and unreliable. Data acquisition and processing, refinement, and validation statistics are summarized in Table S1.

10.1128/mbio.00156-22.1 FIG S1 Data collection and reconstruction of the capsid. (A) Cryo-EM raw image of CtenDNAV-II. (B) Cryo-EM 3D reconstruction of the CtenDNAV-II capsid. The capsid is viewed down the 5-fold axis and radially colored from blue to red. The icosahedral 5-fold, 3-fold, and 2-fold axes are labelled as 5, 3, and 2, respectively. (C) The gold standard FSC resolution curves of masked (blue) and unmasked (green) reconstructions of the CtenDNAV-II capsid. Possible effects of the masking were compensated for by noise randomization (red), to create the final FSC curve (black). The resolution at which the correlation drops below the FSC = 0.143 (gold standard threshold) (25, 26) is 2.4 Å. (D) Local resolution of the final reconstruction determined by Relion. Left: a histogram created by Relion showing how many voxels have a given local resolution, and the middle and right panel shows the reconstruction colored from red to white to blue that corresponds to local resolutions 2.6, 2.4, and 2.2, respectively. The icosahedral 5-fold, 3-fold, and 2-fold axes are labeled as 5, 3, and 2, respectively, in the middle. The front half of the reconstruction is removed at right to visualize the inside of the capsid. (E) Refined side chains of representatives of secondary structural elements and areas that differ between the three subunits. Download FIG S1, PDF file, 2.8 MB. Copyright © 2022 Munke et al. 2022 Munke et al. https://creativecommons.org/licenses/by/4.0/ This content is distributed under the terms of the Creative Commons Attribution 4.0 International license.

10.1128/mbio.00156-22.2 FIG S2 Data processing of outer genome layer. (A) 3D classes from Relion generated during capsid reconstruction indicated that the capsid interior possessed higher order structure. (B) FSC curves of the outer layer where the curves and resolution estimate of 13 Å at FSC = 0.143 were generated by Relion. Due to the occasionally noisy curve, the final corrected curve in black at FSC = 0.143 and 0.5 are shown as zoomed in views in the insets, where the circles indicate the two closest *x*-coordinates. (C) 2D classes generated by cryoSPARC (82) after subtracting the signal of the capsid and genome core. (D) The central spool with three turns and an approximate model of DNA containing 630 bp. Download FIG S2, PDF file, 1.9 MB. Copyright © 2022 Munke et al. 2022 Munke et al. https://creativecommons.org/licenses/by/4.0/ This content is distributed under the terms of the Creative Commons Attribution 4.0 International license.

10.1128/mbio.00156-22.8 TABLE S1 Data collection and processing (A) and structure refinement and validation of capsid model (B). Download Table S1, DOCX file, 0.01 MB. Copyright © 2022 Munke et al. 2022 Munke et al. https://creativecommons.org/licenses/by/4.0/ This content is distributed under the terms of the Creative Commons Attribution 4.0 International license.

### Different conformations of capsid proteins within the asymmetric unit.

**FIG 1:**
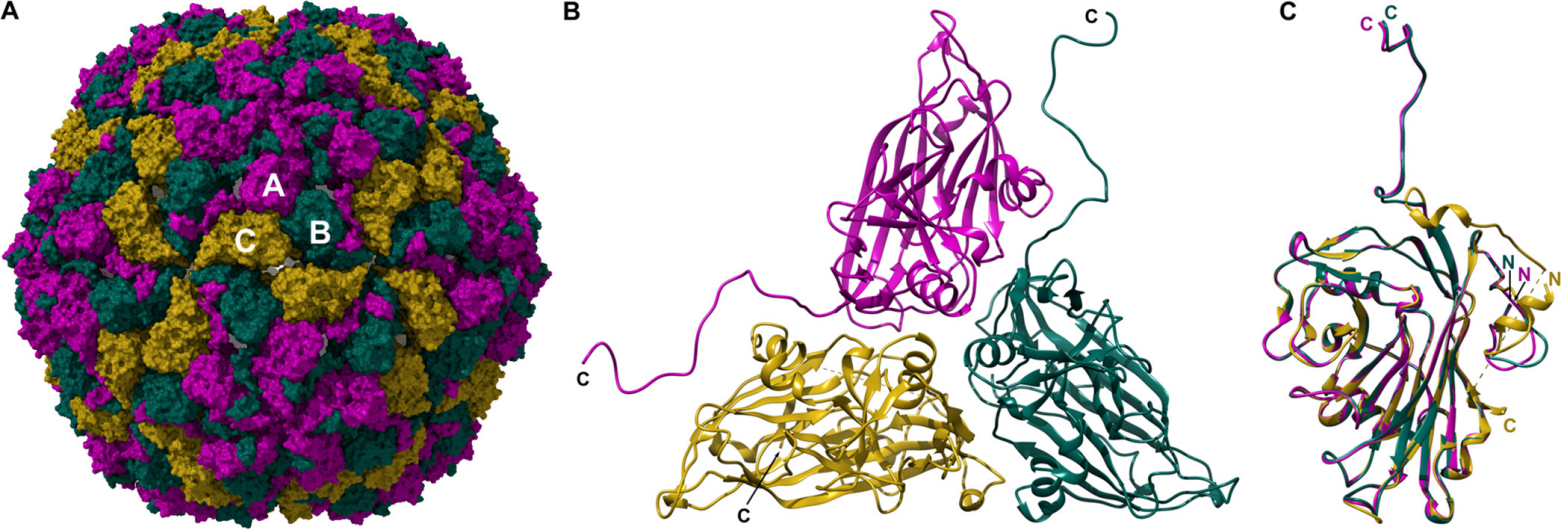
Atomic model of the CtenDNAV-II capsid. The three subunits A, B, and C are colored purple, green, and yellow, respectively, in panels A to C. (A) The entire capsid rendered with a surface representation viewed down an icosahedral 2-fold axis. (B) The secondary structure of one single asymmetric unit viewed from the outside. (C) Superimposition of the three subunits.

**FIG 2:**
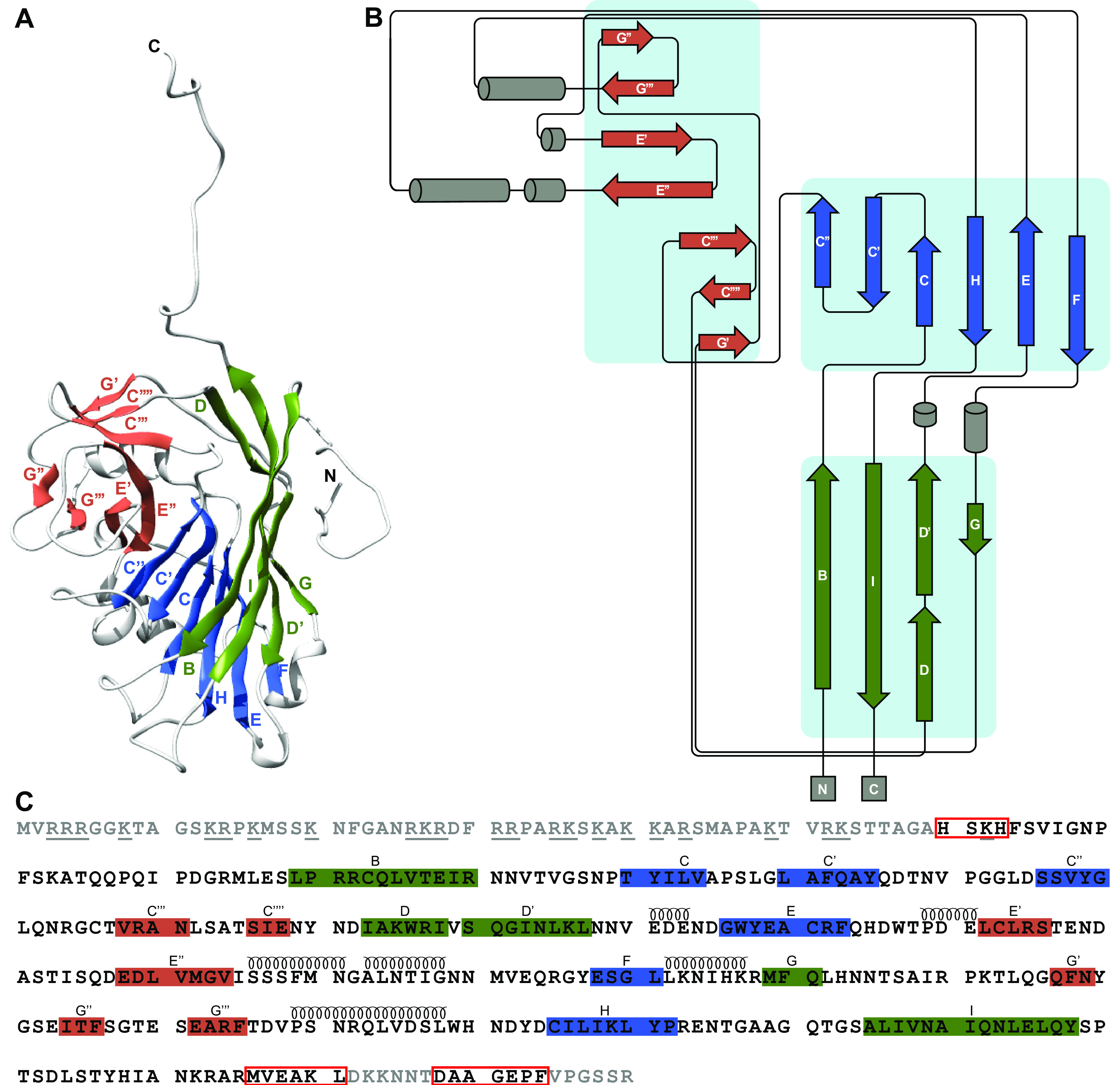
Capsid protein topology and structural organization. The β-strands are colored according to which β-sheet they belong to. The β-strands within the green and blue β-sheets are named alphabetically according to the conventional jelly-roll fold nomenclature (B to I). (A) The secondary structure of subunit A. (B) Schematic diagram of the secondary structure. (C) The amino acid sequence of the CtenDNAV-II capsid protein, starting from residue 1. Each line has 70 residues and is further subdivided into blocks of 10 residues by spaces within the sequence. The residue numbering is the same as in PDB entry 7NS0. Modeled and unmodeled residues are colored black and gray, respectively. Residues highlighted with red rectangles were partly unmodeled: H60 to H63 in subunits A and B, M366 to L377 in subunit C, and D378 to F384 in subunits A and B. The assigned secondary structure is shown schematically above the sequence. The underlined residues in the unmodeled N terminus indicate the numerous positively charged residues.

The CtenDNAV-II capsid displays T = 3 symmetry, i.e., 180 capsid protein protomers assemble such that the asymmetric unit comprises 3 capsid subunits in 3 quasiequivalent positions termed A, B, and C (Fig. 1A and B). For subunit A and B, residues 64 to 371 were modeled, and for subunit C, residues 60 to 365 and 378 tp 384 were modeled (Fig. 2C). The A and B subunits were close to identical (Fig. 1C): the C terminus of the A and B subunits form long tails that end on the capsid surface around the 3- and 5-fold axes, respectively. Here, the last 19 residues could not be modeled (Fig. 2C); however, additional density was visible in the map when the contour level was decreased (Fig. S3), indicating that the C terminus forms flexible protrusions on the capsid surface. The termini of subunit C differs from the A and B subunits (Fig. 1C). Contrary to the long tail toward the capsid surface, the C terminus of the C subunit is directed toward the capsid interior (Fig. 1C and 6). The N termini of all subunits are presumably located on the capsid interior; however, the first 59 to 63 residues could not be modeled (Fig. 2C). Numerous positively charged residues can be found in the N terminus, thus possibly interacting with the genome. Residues 366 to 385 were only partially modeled, and the last residues (386 to 390) were unmodeled in all subunits (Fig. 2C), suggesting different conformations and/or flexibility. The N terminus of the C subunit has a small α-helix on the capsid interior that was not present in the other two subunits (Fig. 1C). The modeling of the C subunit was aided by a structure prediction (Fig. S4) from the AlphaFold package (29). The termini of the predicted model were more similar to the C subunit than to the A and B subunits. The N terminus of the AlphaFold model was confidently predicted (pLDDT >90) from H60, which was also the first residue of the C subunit that could be confidently modeled based on the cryo-EM map (Fig. 2C). As expected, based on the experimental data, the first 59 residues of the N terminus of the predicted structure are disordered. The C subunit was confidently modeled based on the cryo-EM map until R365 (Fig. 2C). With guidance from the AlphaFold model, another seven residues (D378 to F384) could be modeled. Residues 366 to 377 could not be confidently modeled based on the experimental data and are thus not included in the final model (PDB: 7NS0); however, based on the appearance of the cryo-EM density and the predicted model that has a pLDDT score ranging between 77 and 93 in this part, an α-helix is likely located there (Fig. S4). The modeled termini and corresponding cryo-EM map are displayed in Fig. S1E.

10.1128/mbio.00156-22.3 FIG S3 Unmodeled density on the capsid surface. Cryo-EM 3D reconstruction of the CtenDNAV-II capsid at low contour level. The map (originally gray) was colored according to the model using the command “color zone” in Chimera X with a color distance of 6 Å and the same color code as Fig. 1 (subunit A, purple; subunit B, green; and subunit C, yellow). Unmodeled density from the C-terminal ends of subunit A and B that remained gray after zone coloring is visualized around each 3- and 5-fold axis, for which examples are indicated with arrows and dashed circles. The positions of the 5-fold, 3-fold, and 2-fold axes are shown by a white pentamer, triangle, and ellipse, respectively. Download FIG S3, EPS file, 2.7 MB. Copyright © 2022 Munke et al. 2022 Munke et al. https://creativecommons.org/licenses/by/4.0/ This content is distributed under the terms of the Creative Commons Attribution 4.0 International license.

10.1128/mbio.00156-22.4 FIG S4 Comparison between the experimentally determined model of the C subunit and the AlphaFold predicted model. Left: the experimentally determined model of the C subunit; right: the computationally predicted structure of the CtenDNAV-II capsid protein using the AlphaFold package. The predicted model is colored red to blue according to the pLDDT score. Download FIG S4, EPS file, 1.8 MB. Copyright © 2022 Munke et al. 2022 Munke et al. https://creativecommons.org/licenses/by/4.0/ This content is distributed under the terms of the Creative Commons Attribution 4.0 International license.

### Unique features of the CtenDNAV-II capsid protein jelly-roll fold.

The canonical viral jelly-roll consists of eight anti-parallel β-strands that are named from B to I and arranged in two four-stranded sheets (BIDG and CHEF). The loops connecting each strand are named BC, CD, etc. (30, 31). For CtenDNAV-II, the two sheets are formed by strands BIDD’G and C″C’CHEF, respectively (Fig. 2A to C), thus containing three additional strands (D’, C,’ and C″) compared to the standard viral jelly-roll fold. In addition, a third antiparallel β-sheet with seven strands is intertwined within the jelly-roll, i.e., strands from the third sheet are formed by extensions of loops CD, EF, and GH of the jelly-roll. The third sheet, which is located on the capsid surface, is thus composed of two C-strands (C‴ and C″″), two E-strands (E’ and E″), and three G-strands (G’, G,″ and G‴) (Fig. 2B). Notably, AlphaFold accurately and confidently predicted the jelly-roll fold as well as the fold of the additional β-sheet, which had pLDDT scores of >90 and >70, respectively (Fig. S4). In conclusion, the capsid protein of CtenDNAV-II has three unique features: three additional strands in the jelly-roll fold, an extra surface-exposed β-sheet, and an unusual conformation of the C termini of subunit A and B that extends out from the jelly-roll core as long tails toward the capsid surface.

### Unmodeled density blob in interface between capsid protein subunits.

**FIG 3:**
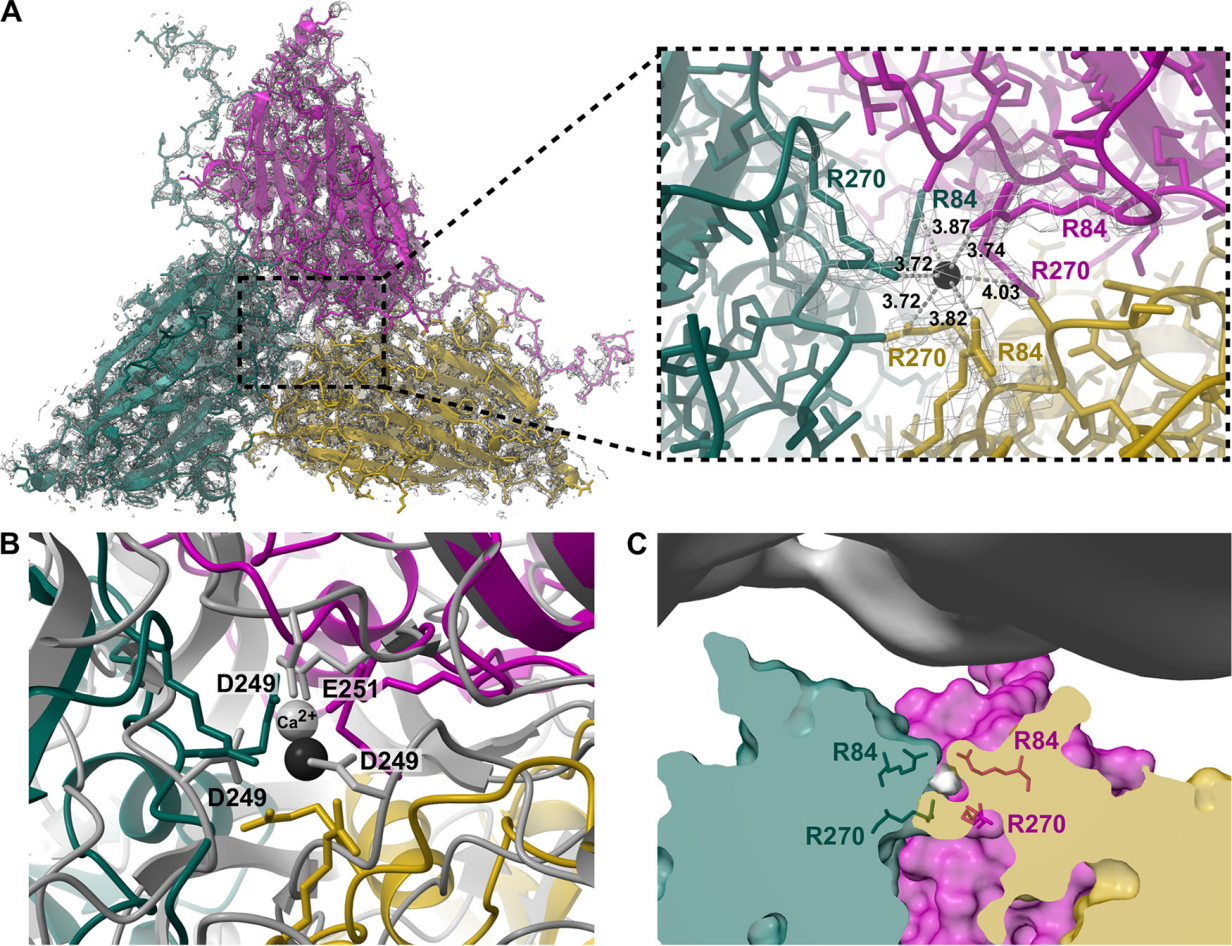
Unassigned density in the subunit interface. (A) The model of a single icosahedral protomer viewed from the inside of the capsid and corresponding cryo-EM map is visualized to the left. To the right is a closeup view of the subunit interface. A marker (black) was placed in the center of the unmodeled density using Chimera X to measure the distances to the surrounding arginine residues. (B) Same view as in panel A, but aligned with Flock House virus (*Nodaviridae*) (PDB: 4FTB). Putative amino acids interacting with Ca^2+^ (gray ball) are labeled and shown as sticks. (C) A clipped side view of an icosahedral protomer with unmodeled density in gray surrounded by arginine residues. The map from the outer genome layer is shown in the top.

An unmodeled density blob was visible in the interface between the three subunits that constitute one protomer (Fig. 3A). Metal ions, commonly Ca^2+^ (32, 33) but also Zn^2+^ (34), are sometimes found in the interface of capsid protein subunits and have shown to be important for viron stability. The unmodeled density in the subunit interface of CtenDNAV-II has a pyramidal to spherical shape (Fig. 3C) and is surrounded by six arginine residues (R84 and R270) that resemble an octahedral coordination geometry (Fig. 3A). The distance from the center of the density to surrounding nitrogen atoms on the arginine sidechains is 3.7 to 4.0 Å. The long distance and the fact that arginine residues would be in the coordination sphere suggest that a metal ion at this position is unlikely (35). However, the blob is the last density that remains when the contour level is increased, which strongly suggest that it is a metal ion. A calcium ion has been reported in nodaviruses, such as Pariacoto virus and Flock House virus (36), at a close to identical location (2.6 Å apart) to the unmodeled blob in CtenDNAV-II (Fig. 3B), which is interesting from an evolutionary perspective, as described later. Considering the location of the unmodeled density, close to the inside of the capsid (Fig. 3C), and the positively charged arginine sidechains, another possibility could be that the density blob originates from a piece of genome. However, the unmodeled blob appears isolated and structurally obstructed by R84 from the capsid interior (Fig. 3C). A multiple sequence alignment (Fig. S5) shows that a positively charged residue (either arginine or lysine) is rather common (16 out of 24 sequences) among bacilladnaviruses at the corresponding position of R84. The second arginine, R270, is very unusual at the corresponding position; however, several sequences have a histidine one amino acid upstream, which potentially could serve the same function. Future research will have to discern the true nature of the blob.

10.1128/mbio.00156-22.5 FIG S5 Multiple sequence alignment with the CtenDNAV-II capsid protein. Selected regions from a multiple sequence alignment performed on sequences resulting from a BLAST search with default search parameters. The sequence identities to the CtenDNAV-II query sequence are listed to the right. Residues aligned with R84 and R270 are highlighted in bold. Download FIG S5, EPS file, 0.1 MB. Copyright © 2022 Munke et al. 2022 Munke et al. https://creativecommons.org/licenses/by/4.0/ This content is distributed under the terms of the Creative Commons Attribution 4.0 International license.

### Structure-based phylogeny reveal evolutionary relationship to ssRNA viruses.

Previous structures of so-called CRESS DNA viruses (phylum *Cressdnaviricota*) (18) include viruses from families *Ciroviridae* (e.g., 3R0R and 5ZJU), *Geminiviridae* (e.g., 6F2S and 6EK5), and *Nanoviridae* (6S44), whose capsids follow T = 1 symmetry. The capsid proteins of these three families also contain a jelly-roll domain but lack the third surface-exposed β-sheet and C-terminal tail found in CtenDNAV-II. The DALI web server (37) was used as an initial step to investigate the previous theory that ssDNA viruses have acquired their capsid protein from ssRNA viruses (6, 21). Indeed, the result indicated that the CtenDNAV-II capsid protein is more similar to capsid proteins of ssRNA viruses than to other ssDNA viruses (Table S2). The closest ssDNA virus was that of Beak and feather disease virus (*Circoviridae*), which ended up oin 11th place (z-score 8.7) behind 10 RNA viruses. Highest similarity was found between CtenDNAV-II and ssRNA viruses from families *Carmotetraviridae*, *Alphatetraviridae*, and *Nodaviridae*, which had z-scores of 14.4 to 15.3 (Table S2). Notably, only nodaviruses from the genus *Alphanodavirus* were included and none from *Betanodavirus* or the informal group of gamma-nodaviruses. Z-scores below 2 are considered insignificant, scores between 2 and 8 are a gray zone, scores between 8 and 20 indicate that two structures probably are homologous, and with a z-score above 20 they are definitely homologous. For an in-depth explanation of the DALI method and z-scores, see Holm (38).

10.1128/mbio.00156-22.9 TABLE S2 Table of viruses used for structural comparison. Download Table S2, EPS file, 0.2 MB. Copyright © 2022 Munke et al. 2022 Munke et al. https://creativecommons.org/licenses/by/4.0/ This content is distributed under the terms of the Creative Commons Attribution 4.0 International license.

**FIG 4:**
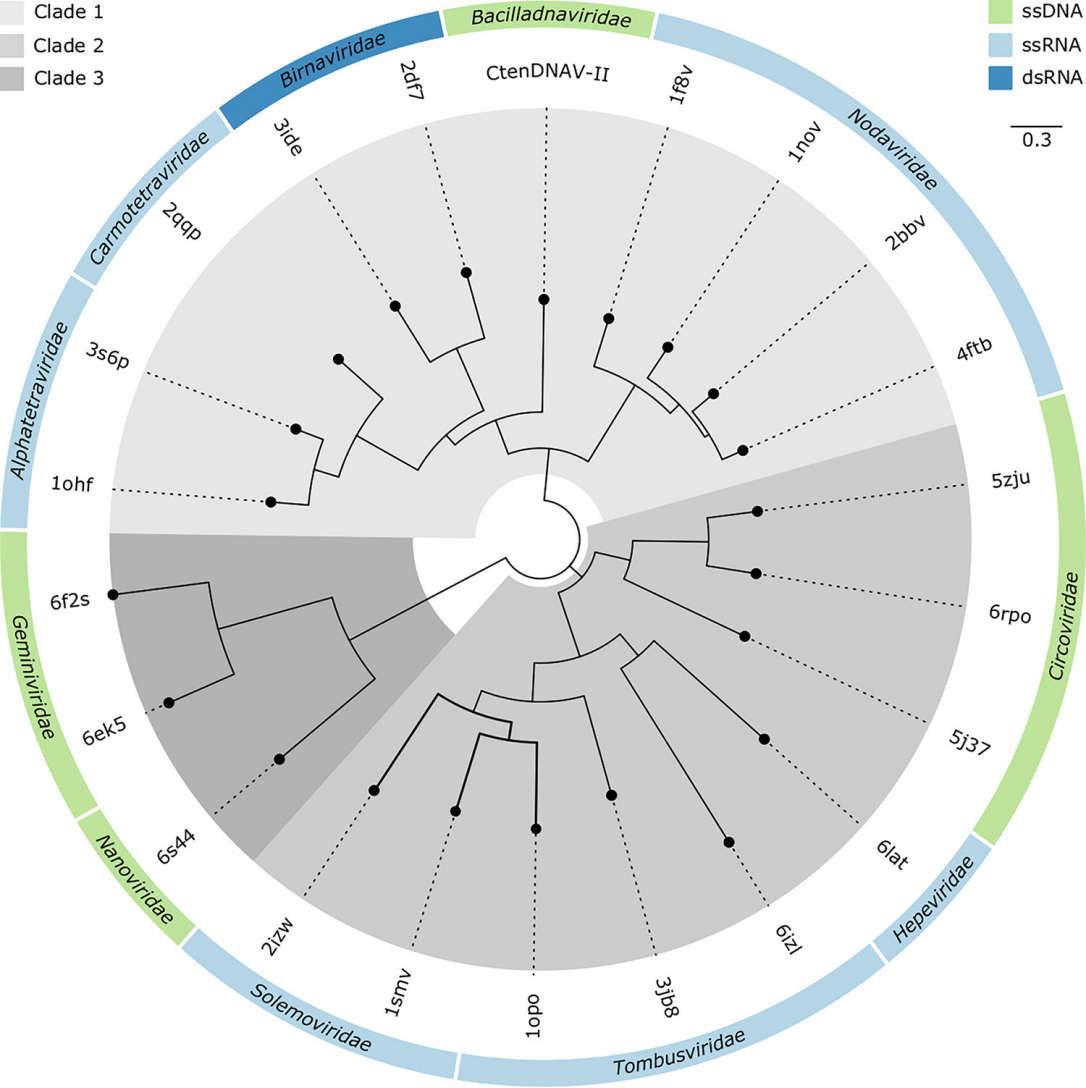
Structure-based phylogenetic tree of the jelly-roll folds. The three major clades are highlighted in different shades of gray. The tree is reconstructed based on RMSD values from superpositions performed on the common jelly-roll fold core. The chain identity and residues included for each structure are listed in Table S2. A phylogenetic tree on full-length chains is shown in Fig. S6A. The RMSD tables of the jelly-roll fold and full-length superpositions are available in Data Set S1.

A structure-based phylogenetic analysis was used to further investigate the relationship between the capsid proteins of CtenDNAV-II and ssRNA viruses. In summary, structures with DALI-scores >8 and three additional CRESS DNA viruses were used together with the CtenDNAV-II capsid protein structure as inputs to the program MUSTANG (39). A complete list of structures used for the analysis is shown in Table S2. The root mean square deviation (RMSD) values provided by MUSTANG were then used to create phylogenetic trees (Fig. 4 and Fig. S6A). The RMSD matrices created by MUSTANG that were used to create the phylogenetic trees are available in Data Set S1. The compared structures have various polypeptide lengths, ranging from 172 amino acids for Faba bean necrotic stunt virus (*Nanoviridae*) to 644 amino acids for Nudaurelia capensis omega virus (*Alphatetraviridae*), and thus the longer structures e.g., from CtenDNAV-II, carmotetraviruses, alphatetraviruses, nodaviruses, and birnaviruses have additional folds and domains in addition to their jelly-roll fold core (Fig. S7). The phylogenetic analysis was therefore performed using only the jelly-roll fold core, for which the result is presented in Fig. 4. The selected amino acid numbers for each structure in the jelly-roll fold comparison are listed in Table S2. Figure 4 displays three clades with different ssDNA viruses in each clade. CtenDNAV-II is placed together with RNA viruses from families *Nodaviridae*, *Alphatetraviridae*, *Carmotetraviridae*, and *Birnaviridae* (Clade 1), thus corroborating the result from the DALI search (Table S2) as well as the previous HGT theory by Kazlauskas et al. (6). The ssDNA circoviruses were organized in a second clade together with ssRNA viruses from families *Tombusviridae*, *Hepaviridae*, and *Solemoviridae*, whereas the other two ssDNA families *Geminiviridae* and *Nanoviridae* were placed alone in the third clade. As a comparison, the phylogenetic analysis was also performed by using full-length chains (Fig. S6A). The result is to some extent similar: CtenDNAV-II and nodaviruses still share structural similarities, and CtenDNAV-II and other ssDNA viruses are organized into different clades; however, the details in the trees differ between the two approaches. For example, only CtenDNAV-II and the nodaviruses appear related when using the full-length chains, whereas carmotetraviruses, alphatetraviruses, and birnaviruses are placed in the other clades. However, our opinion is that Fig. 4 better represents the evolutionary relationship between these viruses, since the jelly-roll fold, which is the conserved structural core and a better representation of the primordial fold, is in some cases misaligned when using the full-length chains (see example in Fig. S6B).

10.1128/mbio.00156-22.6 FIG S6 Complementary information on the phylogenetic analysis. (A) Phylogenetic tree using full-length chains. The three major clades are highlighted in different shades of gray. The tree is reconstructed based on RMSD values from superpositions performed on full-length chains. The chain identity for each structure is the same as for Fig. 4 and is listed in Table S2. (B) MUSTANG superposition of CtenDNAV-II and the *Carmotetraviridae* virus (PDB: 2QQP). Comparison between superpositions of jelly-roll folds (left) and full-length chains (right). Secondary structure outside of the jelly-roll fold core are transparent in the right image. Download FIG S6, TIF file, 1.2 MB. Copyright © 2022 Munke et al. 2022 Munke et al. https://creativecommons.org/licenses/by/4.0/ This content is distributed under the terms of the Creative Commons Attribution 4.0 International license.

10.1128/mbio.00156-22.7 FIG S7 Comparison of capsid proteins in Clade 1, colored according to domain. Representatives of capsid proteins in Clade 1 viewed from the side with surface and interior structures on top and bottom, respectively. The surface domains, jelly-roll fold domains and interior/α-helical domains are colored purple, green and yellow, respectively. Download FIG S7, EPS file, 1.5 MB. Copyright © 2022 Munke et al. 2022 Munke et al. https://creativecommons.org/licenses/by/4.0/ This content is distributed under the terms of the Creative Commons Attribution 4.0 International license.

10.1128/mbio.00156-22.10 DATA SET S1 RMSD matrix created by MUSTANG based on a multiple superposition of jelly-roll folds (A) and full-length chains (B). Download Data Set S1, TXT file, 0.00 MB. Copyright © 2022 Munke et al. 2022 Munke et al. https://creativecommons.org/licenses/by/4.0/ This content is distributed under the terms of the Creative Commons Attribution 4.0 International license.

The results presented support the theory of an acquisition of the capsid protein gene in a bacilladnavirus ancestor from a ssRNA noda-like virus via a HGT event, thus supporting the so-called RNA-to-DNA jump scenario for the origin of DNA viruses with jelly-roll capsid proteins (40). Other alternative scenarios include the independent emergence (i.e., convergent evolution) and the gradual transition scenarios (40). While the latter two cannot be completely ruled out, the first scenario is strongly supported for DNA jelly-roll fold containing viruses, for example, due the fact that several different DNA viruses, including bacilladnaviruses, apparently contain capsid proteins that are more similar, on both sequence and structural level, to different RNA viruses, while their rolling-circle replication proteins are polyphyletic (6, 13, 21–23, 41). Findings supporting that ssRNA and ssDNA recombination is plausible include the fact that nucleic acid packing can be unspecific (21, 42) and that single-stranded genomes have similar persistence lengths (43). For more detailed discussions on this topic, see Holmes (44), Krupovic (40), and Moreira and López-García (45).

### Putative emergence of ssRNA, dsRNA, and ssDNA viruses.

**FIG 5:**
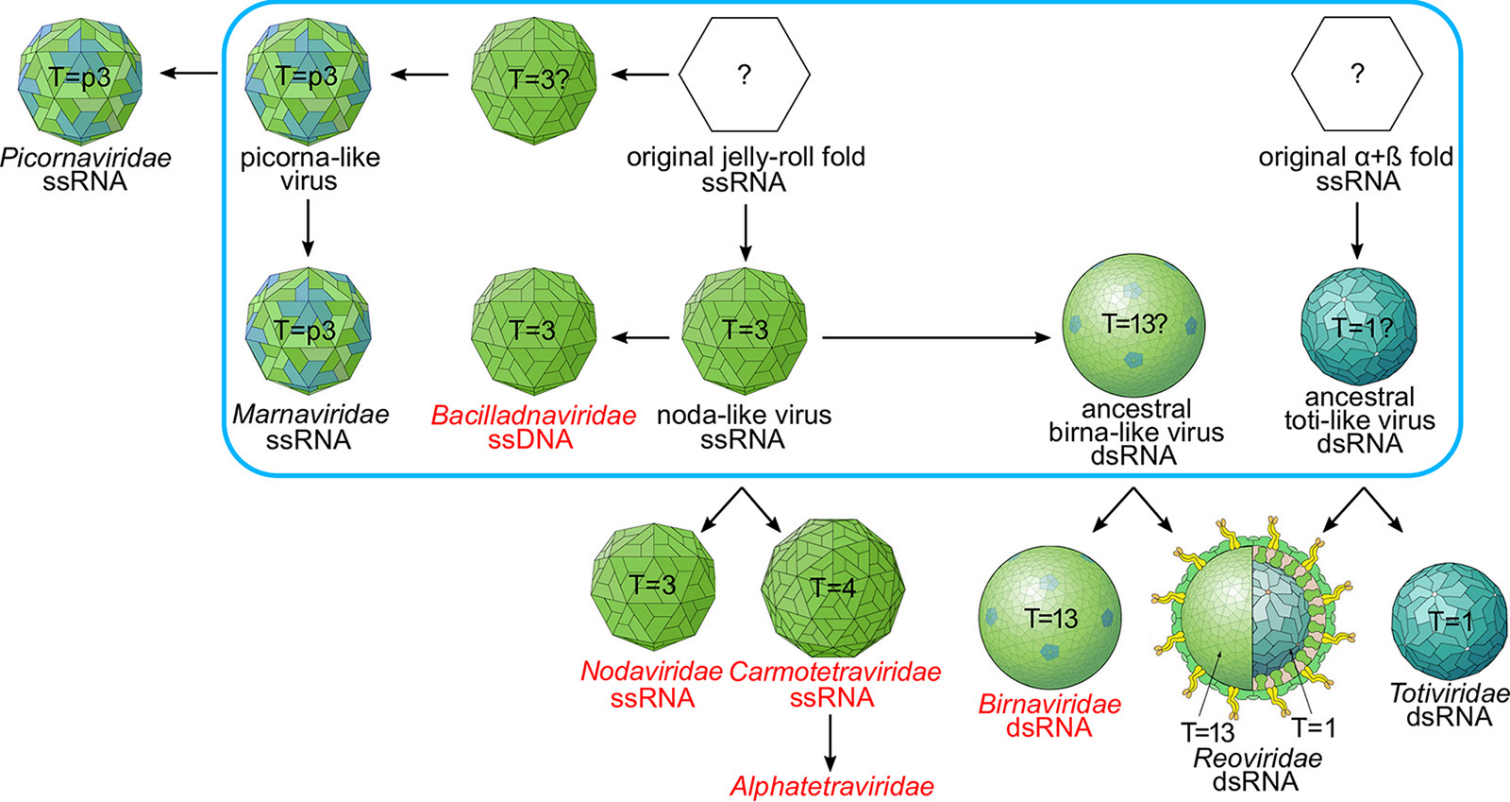
A scheme depicting the putative emergence of virus capsids carrying different nucleic acid genomes. The figure illustrates, based on current knowledge and hypotheses, the most relevant virus groups and the evolutionary relationship between their virus capsids. Viruses in Clade 1 (Fig. 4) are highlighted in red. Events that likely took place at an early stage of evolution in ancient algal pools are circled in blue. The figure is based on results described in this paper as well as previous results: the relationship between bacilladnaviruses, nodaviruses, carmotetraviruses, alphatetraviruses, and binaviruses is described herein as well as in references 6, 21, 46, 47; the relationship between the dsRNA viruses is described in reference 46; the evolution of picornaviruses were described in reference 8; the link between the ssRNA, ssDNA, and dsDNA is described in references 15, 19, 21; and the presence of birna-like and toti-like viruses in the oceans is described in references 78–80. The viron pictures were derived from ViralZone, SIB Swiss Institute of Bioinformatics (81) (https://viralzone.expasy.org/) licensed under a Creative Commons Attribution 4.0 International License.

The organization of ssDNA viruses into three different clades supports a polyphyletic origin of ssDNA viruses and the independent acquisition of capsid protein genes from different RNA viruses (21). Our findings (Fig. 4) also support the previously described close evolutionary relationship between virus families *Carmoteraviridae*, *Alphatetraviridae*, *Nodaviridae*, and *Birnaviridae* (21, 46, 47). The capsid proteins of the T = 4 tetraviruses have been proposed to be derived from the T = 3 nodaviruses, and in particular, there is a higher degree of similarity between carmotetraviruses and nodaviruses than between alphatetraviruses and nodaviruses, suggesting that carmotetraviruses are closer than alphatetraviruses to the primordial T = 4 virus (47). The dsRNA birnaviruses (T = 13) have been suggested to have a noda/tetravirus-like ancestor (46), which exemplifies the evolutionary link between ssRNA and dsRNA viruses. In turn, an ancestral birna-like virus could have been the precursor of the *Reoviridae* outer capsid layer, whereas the inner layer that has T = 1 symmetry, might have been acquired from an ancestral toti-like virus (46). To put our new structural insights into a broader context, we have updated the putative emergence of viruses harboring different genomes types (Fig. 5).

### Acquired and retained structures in capsids of CtenDNAV-II and ssRNA viruses.

**FIG 6:**
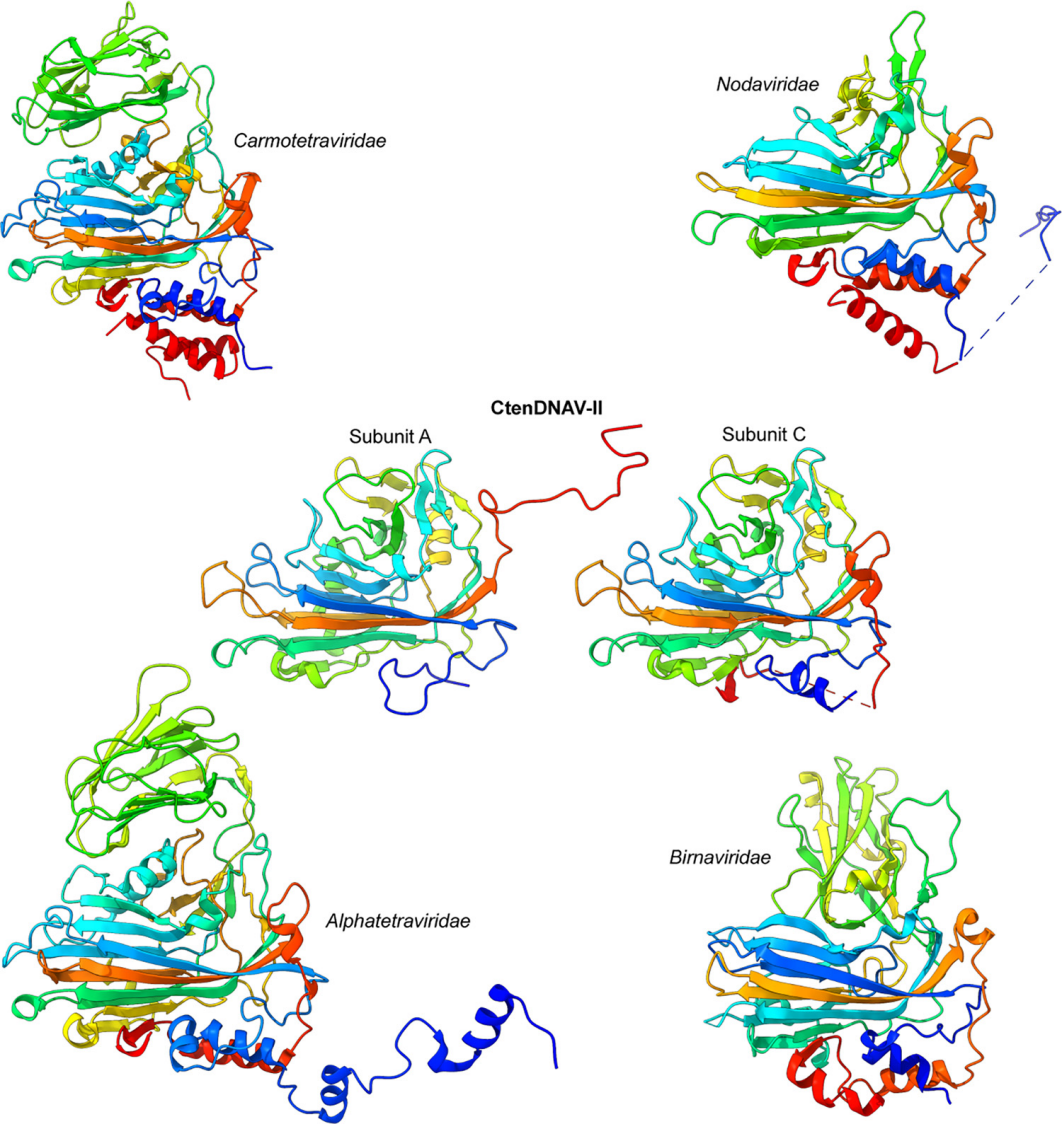
Comparison of capsid proteins in Clade 1. Representatives of capsid proteins in Clade 1 viewed from the side with surface and interior structures on top and bottom, respectively. The chains are colored as rainbow from blue (N terminus) to red (C terminus). Figure S7 shows the same images, but colored according to domain.

The jelly-roll fold of CtenDNAV-II aligns well with the other jelly-roll folds in Clade 1, with RMSD values of 1.4 to 1.7, compared to the jelly-roll folds in the other clades that had RMSD values of 2.2 to 3.6 (Data Set S1). As expected, the differences between the capsid proteins from CtenDNAV-II and carmotetraviruses, alphatetraviruses, nodaviruses, and birnaviruses lie primarily in surface exposed areas and to some extent in the regions facing the capsid interior (Fig. 6 and Fig. S7), i.e., structural elements that have evolved based on the host and on genome packing requirements, respectively. All capsid proteins in Clade 1 have specific surface structures in addition to the jelly-roll fold (Fig. 6 and Fig. S7). While the surface structures of CtenDNAV-II and nodaviruses are formed by extensions of loops in the jelly-roll, carmotetraviruses, alphatetraviruses, and birnaviruses instead have larger separate domains (called Ig-like and P-domains) inserted between two jelly-roll strands (Fig. 6). Unique for CtenDNAV-II is the long C-terminal tail in subunits A and B that extends to the capsid surface, while the C terminus of the capsid protein from the other viruses can be found on the capsid interior similarly as the C terminus of the C subunit in CtenDNAV-II (Fig. 6). The acquired surface features for each virus family (Fig. S7), such as the Ig-like domain of tetraviruses and the P domain of birnaviruses, have been described as putative receptor-binding domains of those viruses (46–50). The unique surface features of CtenDNAV-II include the third β-sheet and the long C-terminal tails of the A and B subunits. Similar to RNA viruses, these acquired features of CtenDNAV-II could potentially be important for host recognition; however, future studies will have to confirm this theory.

Carmotetraviruses, alphatetraviruses, nodaviruses, and birnaviruses have α-helices located on the capsid interior that are formed by the capsid protein termini, which in some structures have been shown to interact with the viral RNA (47). Of the three CtenDNAV-II subunits, the C subunit shows highest similarity to the capsid proteins of the other viruses in Clade 1: both termini are located on the capsid interior and the termini forms α-helices (Fig. 6). The model of the C subunit has one α-helix in the N terminus; however, based on the cryo-EM map and the predicted model by AlphaFold, also the C terminus is likely to form an α-helix (Fig. S4). Superpositions of the capsid proteins from families *Carmotetraviridae*, *Alphatetraviridae*, *Nodaviridae*, and *Birnaviridae* with the predicted model of the CtenDNAV-II capsid protein show that the two predicted terminal α-helices align well with α-helices from the experimentally determined structures. More and longer helices are found among the RNA viruses (Fig. 6 and Fig. S7), and the two below the C subunit could thus be remnants from the noda-like virus ancestor, whereas the C terminus in the A and B subunits has evolved to form other structural elements on the capsid surface as a host adaptation. Thus, the C subunit of CtenDNAV-II presumably resembles the primordial bacilladnavirus fold more than subunit A and B do. Finally, an additional similarity is found in the N terminus of CtenDNAV-II and the ssRNA viruses in Clade 1. Due to disorder, up to 63 residues of the highly positively charged terminus could not be modeled for CtenDNAV-II (Fig. 2C). Likewise, the first ~40 to 70 residues, also heavily populated with arginine and lysine residues, are unmodeled for the ssRNA viruses. This is in contrast to the dsRNA birnaviruses, whose N terminus is largely modeled (missing 5 to 10 residues) and less charged. While differences in surface features between the viruses in Clade 1 could reflect variations in host specificity, the differences on the capsid interior could possibly reflect variations in genome organization. Structural information on the genomes of the RNA viruses in Clade 1 is limited to short nucleotide (nt) segments, which have been revealed even when the icosahedral symmetry has not been broken during the structure determination (36, 47). Despite similarities in the capsid interior among viruses in Clade 1, no DNA segments were found inside CtenDNAV-II during the capsid reconstruction, suggesting that noda-, tetra-, and birnaviruses interact more specifically with their genome than CtenDNAV-II does and that their genomes are organized structurally different. Perhaps the observed adaptations of the CtenDNAV-II capsid interior, with fewer and shorted α-helices, were enough to allow packing a genome that interact nonspecifically with the capsid.

### CtenDNAV-II genome is partially spooled.

**FIG 7:**
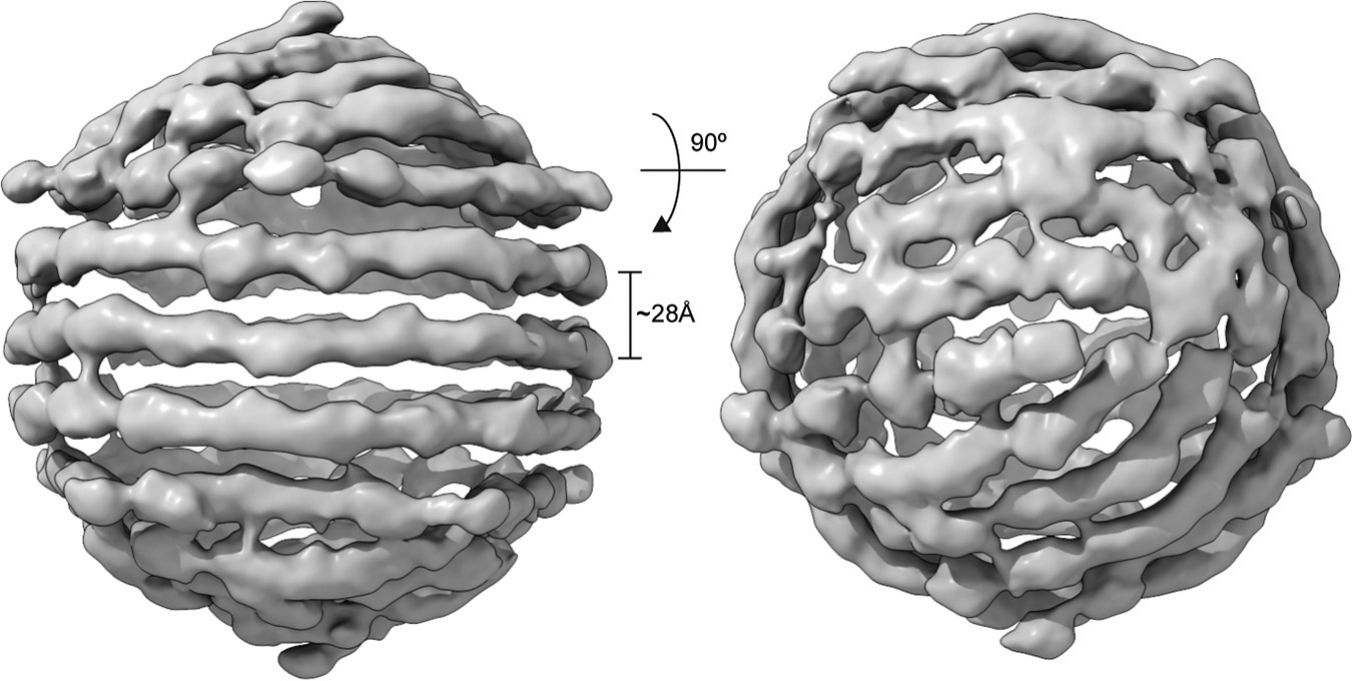
Outer genome layer of CtenDNAV-II. Left: the coil of three turns is visualized in the center of the image, where the individual parallel turns are separated ~28 Å. Right: the image on the left has been rotated 90**°**.

The reconstruction of the outer genome layer (EMDB-12555) displays a coil of three turns (Fig. 7, left), which were readily visible also in 2D classes (Fig. S2C), and on each side of the three turns are additional DNA fragments that do not follow the same spooling arrangement (Fig. 7, right). The spooled genome packaging has previously only been described in viruses with double-stranded genomes (28, 51, 52), whereas the genomes of ssRNA viruses forms secondary structures that organize into branched networks (53–55) or a dodecahedral cage (36, 56).

Considering that spooled genome arrangements have only been observed among viruses with double-stranded genomes and that the distance between the parallel turns are about 28 Å (Fig. 7, left), similar to double-stranded genomes (28, 51, 57), the outer genome layer of CtenDNAV-II could potentially be at least partially formed by double-stranded DNA. In fact, the circular ssDNA genome of CtenDNAV-II includes a partially double-stranded region of 669 bp (17). The low resolution of the outer layer reconstruction prevented precise modeling; however, by manually combining short (5 to 20 nt) A-T base pairs of ideal B-form dsDNA into the density of the spool with three turns, a model with 630 bp could be obtained (Fig. S2D). Thus, hypothetically, the observed spool could correspond to the double stranded region of the genome. However, future bacilladnavirus genome structures at higher resolution will have to confirm present observations and hypotheses as well as if additional base-pairing is formed that produce the semispooling pattern observed on each side of the three central turns.

Previous cryo-EM studies have revealed different types of genome organization for nonenveloped icosahedral viruses that often reflect the physical properties of the genome and the viron assembly mechanisms. Viruses containing double-stranded genomes (dsDNA and some dsRNA) use an NTP-driven motor to form spooled genome structures within a preassembled capsid (28, 51, 52). The spooled genomes are packaged by flexible interactions between the capsid protein and the genome. The interactions are mediated by small contacts with hydrophobic and/or positively charged amino acid residues of their capsid proteins. Segmented dsRNA reoviruses instead form nonspooled or partially spooled genomes with pseudo-D3 symmetry that interact with the RNA-dependent RNA polymerase (57–60). Genomes of ssRNA viruses form secondary structures that can either form a branched network, such as in *Leviviridae* viruses (53–55), or a dodecahedral cage, such as in *Secoviridae* and *Nodaviridae* viruses (36, 56). Contrary to the large double-stranded genomes that arrange within a preformed capsid, the small ssRNA genomes allow a simultaneous and cooperative genome packing and capsid assembly that is governed by specific interactions between the genome and capsid protein (55, 56). Less is known about the genomes of ssDNA viruses; however, similar to viruses with other genome types, the genomes of ssDNA viruses are strongly connected to the packing mechanism (61–63). Some ssDNA viruses, such as phages from the *Microviridae* family, have been shown to combine genome packing properties of both double-stranded viruses and ssRNA viruses by performing genome replication while simultaneously packing the newly synthesized strand into preformed capsids (64). Unlike double-stranded viruses, microviruses do not require additional energy from NTP-driven packaging motors since their genomes do not require as dense packaging (65). Previous structural information on the genomes of ssDNA viruses have been limited to short (<10 nt) nucleotide segments but have revealed specific interactions between the capsid and DNA of single-stranded type (61, 62, 66). The genomes of ssDNA viruses have the capability to form biologically functional secondary structures similar to ssRNA viruses (67) and since the capsid gene of bacilladnaviruses has been horizontally transferred from ssRNA viruses, a similar structural arrangement of the genome as in ssRNA viruses is imaginable also for ssDNA viruses. However, the structure of the CtenDNAV-II genome is much more similar to the spooled genomes found in double-stranded viruses (Fig. 7), thus raising the question whether the CtenDNAV-II capsid preassembles before packing the genome, such as the ssDNA microviruses (64). Interestingly, rod-shaped virus-like particles have been found together with CtenDNAV-II particles in infected host cells and were suggested to be precursors of mature virons (17). Future studies will be needed to unravel the exact mechanisms behind bacilladnaviron assembly and genome packing.

## MATERIALS AND METHODS

### Virus production and purification.

CtenDNAV-II was produced as previously described (17). The crude virus suspension was loaded onto 15% to 50% (wt/vol) sucrose density gradients and centrifuged at 24,000 × *rpm* (102,170 × *g*) for 18 h at 4°C (Sw40Ti rotor; Beckman Coulter). The fractions of the sucrose gradient were applied to SDS-PAGE. The VP2 capsid protein fractions were pooled and subjected to centrifugation at 28,000 rpm (139,065 × *g*) for 3 h at 4°C (Sw40Ti rotor; Beckman Coulter). The pellet was resuspended in 50 mM Tris (pH 7.4), 100 mM NaCl, and 0.1 mM EDTA.

### Cryo-EM and data collection.

An aliquot (3 μL) of purified CtenDNAV-II virons (10 mg mL^−1^) was deposited onto freshly glow-discharged holey carbon-coated copper grids (Quantifoil R 2/2, 300 mesh, copper) followed by 2 s of blotting in 100% relative humidity for plunge-freezing (Vitrobot Mark IV) in liquid ethane. Images were acquired using a Titan Krios microscope (Thermo Fisher Scientific) operated at 300 kV and equipped with a K2 Summit direct electron detector (Gatan) and an energy filter.

### Image processing and 3D reconstruction.

The micrographs were corrected for beam-induced drift using MotionCor2 1.2.6 (68), and contrast transfer function (CTF) parameters were estimated using Gctf 1.06 (69). The RELION 3.1 package (70) was used for particle picking, 2D and 3D classifications, *de novo* 3D model generation and refinement. The capsid reconstruction was further sharpened with DeepEMhancer (71) by using the two half-maps from the refinement. The reconstruction of the capsid was generated in I4 symmetry using 33,507 particles, which were obtained by performing 9 consecutive 2D classification steps. The two genome reconstructions were generated in C1 symmetry using 21,559 particles, which were generated by performing 6 consecutive 3D classifications of the 33,507 particles that were obtained from the 2D classification step. Resolutions were estimated using the gold standard Fourier shell correlation (threshold, 0.143 criterion) (25, 26). The data set and image processing are summarized in Table S1.

To reconstruct the genome without icosahedral symmetry a similar procedure to what has been described by Ilca et al. (28) was carried out. The contribution of the capsid was first subtracted from the map created during the final iteration of the I4 refinement job using the Particle subtraction function in Relion. To create a mask for the particle subtraction, the capsid model was first transformed to a density map using the molmap command in Chimera and then an inverted soft edged mask was created from the density map using relion_mask_create with the –invert option. The subtraction was followed by 3D classification (C1 symmetry), which generated the subset of 21,559 particles that was used for the final C1 refinement. The 3D classifications revealed clear density of an outer layer, and an additional subtraction was therefore performed using a spherical mask of 100 Å before the final refinement. The spherical mask was created using relion_mask_create with the –denovo and –outer_radius options. The two maps (the capsid density created by molmap and the circular map) were combined using Chimeras vop command before creating a new inverted mask using relion_mask_create, which was used for subtraction before the final 3D refinement.

### Model building and refinement.

The atomic model of CtenDNAV-II capsid protein was manually built into the density map using Coot (72). The model was further improved through cycles of real-space refinement in PHENIX (73) with geometric and secondary structure restraints, and subsequent manual corrections by Coot were carried out iteratively. Refinement and validation statistics are summarized in Table S1. The model of the outer genome layer was constructed by manually combining short (5 to 20 nt) A-T base pairs of ideal B-form dsDNA into the density using UCSF Chimera X (74). The structure prediction was carried out using AlphaFold 2.0 (29).

### Structure analysis.

Structural comparison of the CtenDNAV-II capsid protein was initially carried out by the DALI web server as a heuristic search against all structures (as of 2021-07-05) in the PDB (37). Unique viruses (i.e., not unique PDB entries) from the DALI search with z-scores >8 were used for structure-based multiple alignments using the program MUSTANG (39). In addition, three other CRESS DNA viruses with known structure (6S44, 6EK5 and 6F2S) were included in the MUSTANG analysis. Detailed information on the structures used for the comparison can be found in Table S2. The RMSD values provided by MUSTANG (Data Set S1) were used to create phylogenetic trees using the Neighbor-joining method (75) in MEGA X (76, 77), which were further aesthetically modified with FigTree. Figures were prepared using UCSF Chimera X (74).

### Data availability.

The atomic model and map of the CtenDNAV-II capsid was deposited in the PDB (7NS0) and EMDB (12554). The EMDB entry provides the full map, as well as the half-maps, mask, and map sharpened by DeepEMhancer (71). The map of the cryo-EM reconstruction of the outer genome layer was deposited in the EMDB (12555).
