# Supplementary figures and images for: Primordial Capsid and Spooled ssDNA Genome Structures Unravel Ancestral Events of Eukaryotic Viruses
Source: mBio. 2022 Jul 20;13(4):e00156-22. doi: 10.1128/mbio.00156-22 (PMC9426455; doi:10.1128/mbio.00156-22)

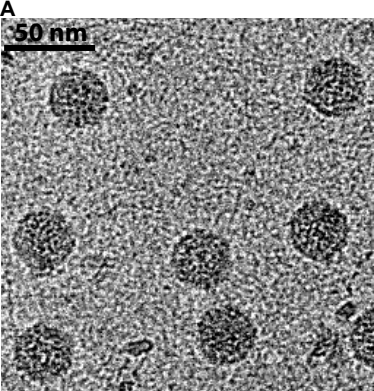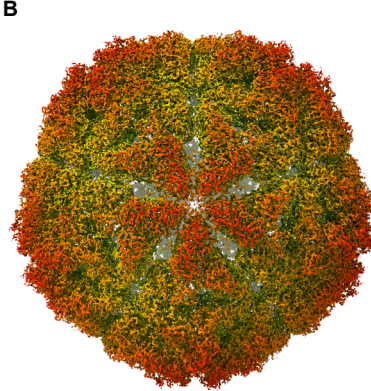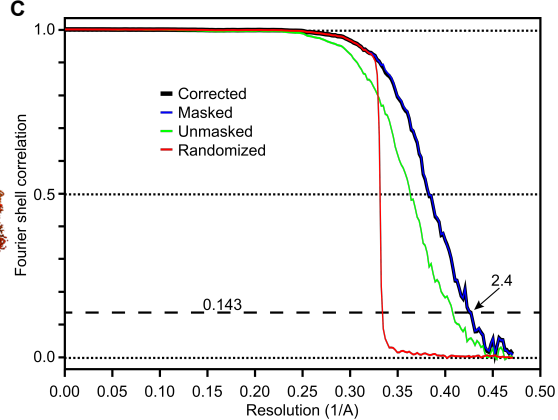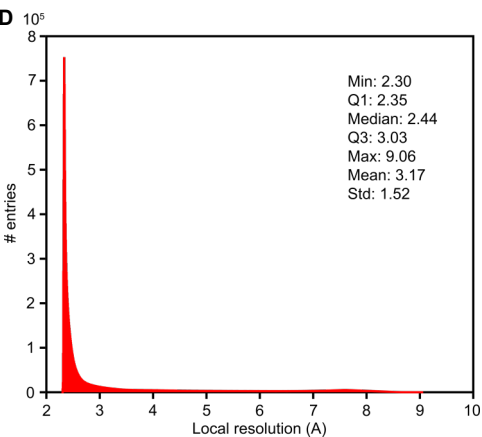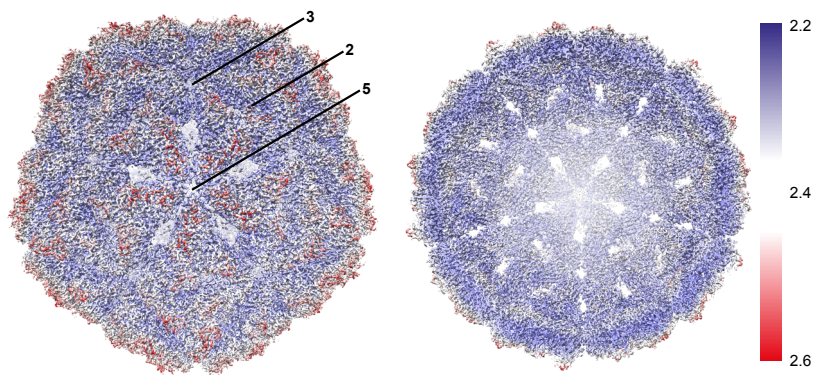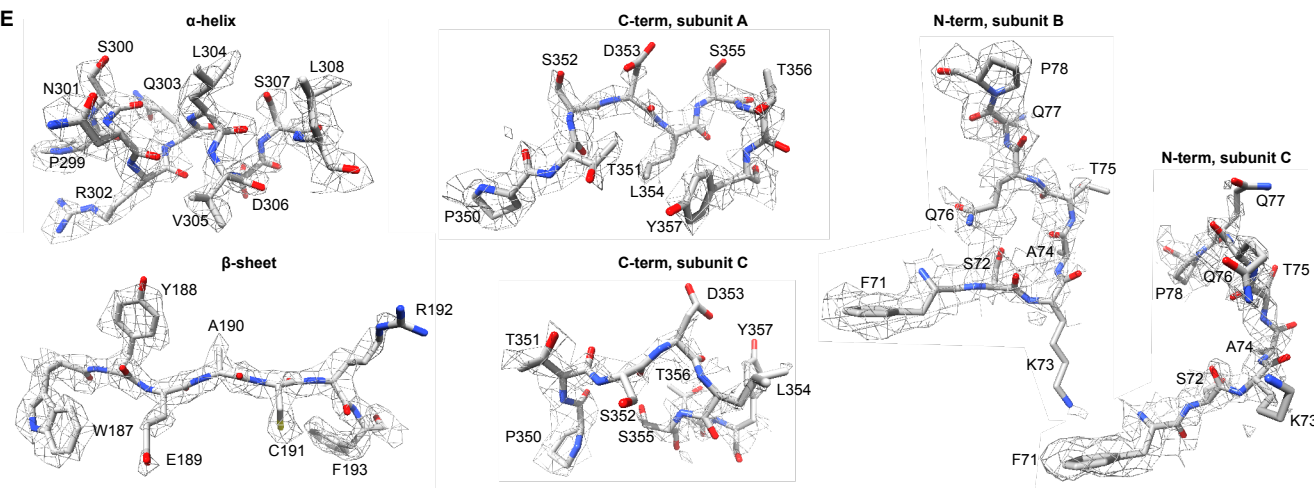

Supplement: FIG S1 [file mbio.00156-22-sf001.pdf]

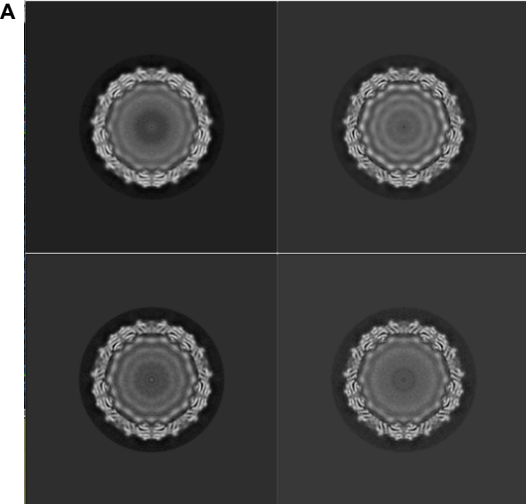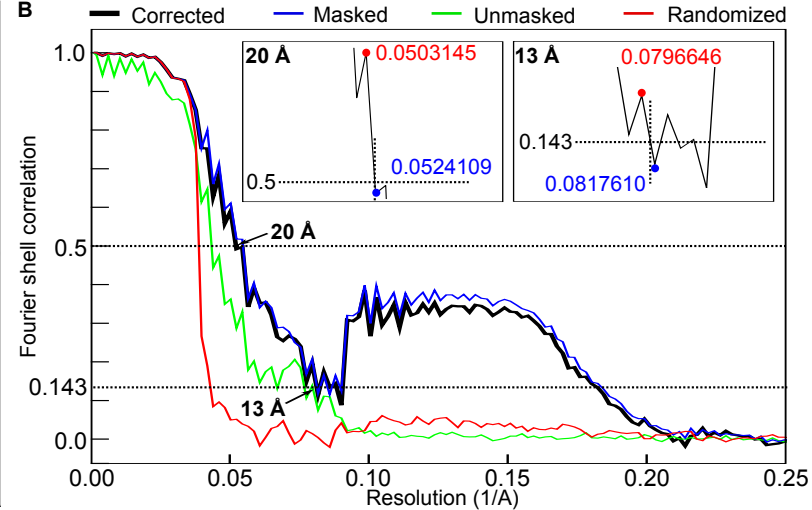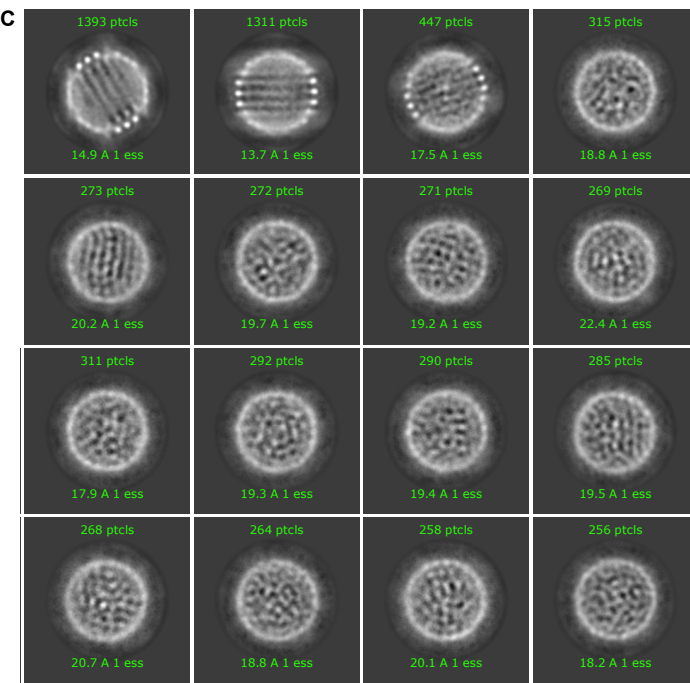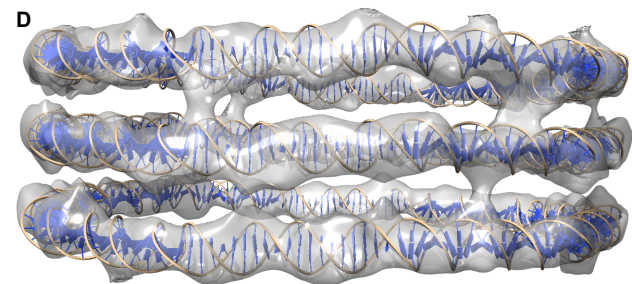

Supplement: FIG S2 [file mbio.00156-22-sf002.pdf]

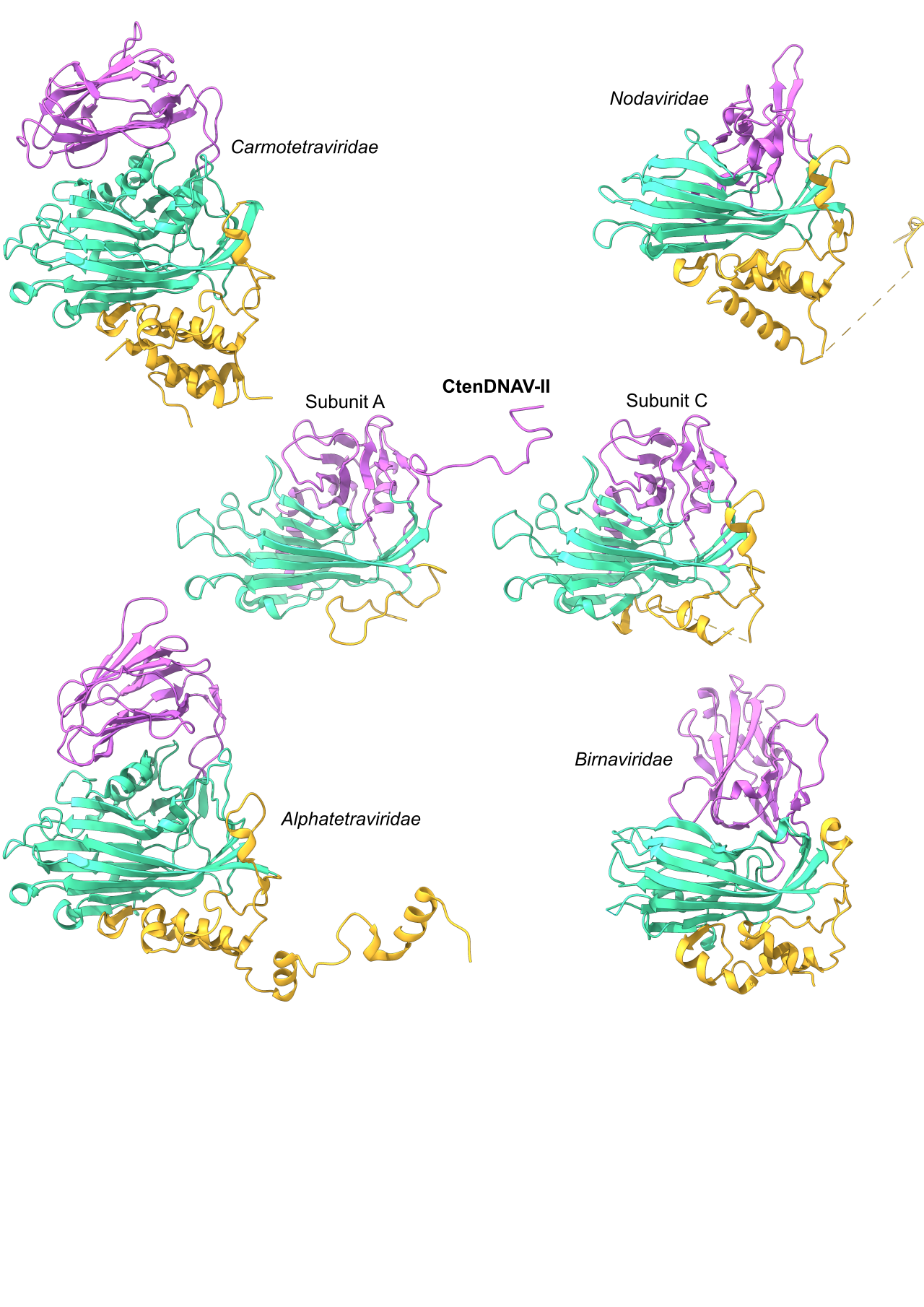

Supplement: FIG S6 [file mbio.00156-22-sf006.tif]
