# Supplementary material for: Primordial Capsid and Spooled ssDNA Genome Structures Unravel Ancestral Events of Eukaryotic Viruses
Source: mBio. 2022 Jul 20;13(4):e00156-22. doi: 10.1128/mbio.00156-22 (PMC9426455; doi:10.1128/mbio.00156-22)
Supplement: TABLE S1 [file mbio.00156-22-st001.docx]

**A**

|  | **Capsid**  **EMD-12554** | **Genome – outer layer**  **EMD-12555** |
| --- | --- | --- |
| Magnification | 140,000 | |
| Voltage (kV) | 300 | |
| Defocus range (μm) | -1.00 to -3.00 | |
| Microscope | Titan Krios | |
| Camera | K2 | |
| Total electron dose (e^-^/Å^2^) | 37 | |
| Pixel size (Å) | 1.06 | |
| Final particle number | 33,507 | 21,559 |
| Symmetry imposed | I4 | C1 |
| Map resolution (Å) (FSC=0.143) | 2.3 | 13 |
| Map resolution range (Å) | 2.3-9.1 | N/A |
| Map sharpening B-factor (Å^2^) | -71.462 |  |

**B**

| **Model** |  |
| --- | --- |
| PDB | 7NS0 |
| Composition |  |
| Chains | 3 |
| Atoms | 14212 |
| Protein residues | 929 |
| Bonds (RMSD) |  |
| Length (Å) | 0.006 |
| Angle (°) | 0.717 |
| MolProbity score | 1.48 |
| Clash score | 3.71 |
| Ramachandran plot (%) |  |
| Outliers | 0.00 |
| Allowed | 4.56 |
| Favored | 95.44 |
| Rotamer outliers (%) | 0.99 |
| Cβ outliers (%) | 0.00 |
| CaBLAM outliers (%) | 1.53 |
| B-factors (Å^2^) (min/max/mean) | 4.04/30.20/9.67 |
| **Data** |  |
| d99 masked (full/half1/half2) | 2.2/3.3/3.3 |
| d99 unmasked (full/half1/half2) | 2.1/3.1/3.1 |
| FSC (model) = 0 (masked/unmasked) | 2.0/2.1 |
| FSC (model) = 0.143 (masked/unmasked) | 2.1/2.2 |
| FSC (model) = 0.5 (masked/unmasked) | 2.3/2.7 |
| **Model vs. Data** |  |
| CC (mask) | 0.82 |
| CC (box) | 0.39 |
| CC (peaks) | 0.22 |
| CC (volume) | 0.73 |
| EMRinger score | 6.60 |
